# What Are the Chances That You Will Die Rich?

**Published:** 1919-06

**Authors:** Richard D. Wyckoff


### WHAT ARE THE CHANCES THAT YOU WILL DIE RICH?

BY RICHARD D. WYCKOFF

This question was suggested by a perusal of the first chapter of Mr. George E. Barrett's series on "How to Invest."

At the outset let me say: From the mathematical standpoint, your chances are very poor indeed.

Mr. Barrett submits a very interesting compilation by the American Bankers' Association and various life insurance companies, giving the life experience of one hundred average men who begin life without resources. I have reproduced this in a graph, because it is more understandable and convincing in that form.

At the age of thirty-five, after passing ten years of their business experience, we find that out of one hundred men five are deceased, while sixty out of the hundred are in an improved financial condition, ranging from moderate resources to wealthy.

It is an old saying it is easier to make money than to keep it, and the graph proves this to be a fact, because at the age of forty-five we find that only four out of the hundred are wealthy, and that sixty-five out of the eighty-four then living are self-supporting, but have no other resources. These figures would seem to fully bear out the old adage. Just think of it—out of sixty men, of which forty at the age of thirty-five had moderate resources, ten were in good circumstances, and ten were wealthy, only four remain that may be regarded as wealthy, and none of the others have any resources except their weekly salaries.

From twenty-five to thirty-five they have made money, but from thirty-five to forty-five they were not able to keep it. How I wish that every business man at the age of twenty-five would begin to study the Science of Investment so that by the time he has accumulated a competency he will know how to invest it wisely.

All sorts of traps are laid for those who have money. A thousand mice nibble at every man's fortune. It seems that while he has youth and enthusiasm circumstances favor him, but as the majority in their earlier business years have received no training whatsoever in the care and investment of money, they are unable to retain their foot-hold on the fortunate side of life. By the age of forty-five, 15 per cent. are not self-supporting, and 65 per cent. have practically no resources.

This is really an appalling situation, but it grows worse by the age of fifty-five. Only eighty now remain out of the original hundred, and of these thirty men are more or less dependent upon their children, their relatives, or charity. Forty-six, or over half those living, have been unable to accumulate anything. Only four are either rich or in good circumstances.

At the age of sixty-five, out of sixty-four living, fifty-four are dependent and only six are self-supporting

However, the worst situation of all is found in the statistics at the age of seventy-five. Out of sixty-three who have died, only three left any estate whatsoever; thirty-four are now absolutely dependent upon their children, their relatives or charity.

*Out of the original one hundred men only five left enough in their estates to cover their funeral expenses.*

Such is the history of these hundred average men, and the percentage represented at these different ages shows you just what the chances are that you will die rich.

Does not this make you stop and think?

Do you not see how absolutely vital it is that you should not only learn how to make money, but how to keep it?

Note that out of four men who were wealthy at the age of forty-five, there were still four at fifty-five and sixty-five, but at the age of seventy-five they had been reduced to three. Possibly this is through death—the figures do not show—but there is something in this graph which indicates that when a man reaches the age of forty-five, and has by that time learned to take care of his money, he retains it for the balance of his life. There is no doubt at passing ten years of their business experience, we find that out of all that his length of life is greatly increased by the fact that he is in easy financial circumstances and can supply himself with the comforts of life and not be subjected to the thousand worries that cut many off in their prime.

The lessons which we must draw from these authoritative statistics are these: Learn how to keep your money, once you have made it. Study this problem just as you study the other problems in your business.

Why go through all the thousand motions necessary to accumulate money, and then in the later years of your life, when you need it most, see it drift away from you, or be wheedled away from you by persons who have more interest in their own pockets than in your welfare?

And it is important that you learn how, while you are in your most vigorous years, because the lesson will then be indelibly impressed upon you and will last the balance of your life. Do not leave it until old age, because by that time your judgment will not be half so good.

If you have sons at the age of twenty-five or older, insist upon their learning their investment lesson so that they, too, will be fortified against the dangers that beset a man with money, and so that they and their families may be placed in a different position from the ninety-five out of one hundred who did not leave enough to cover their funeral expenses.—*Dental Facts.*

